# The Russian Conduit - Combining Bentall and Ozaki Procedures for Concomitant Ascending Aorta Replacement and Aortic Valve Neocuspidization

**DOI:** 10.21470/1678-9741-2019-0329

**Published:** 2019

**Authors:** Roman Komarov, Igor Chernov, Soslan Enginoev, Michel Pompeu B. O. Sá, Dmitry Tarasov

**Affiliations:** 1 Clinic of Aortic and Cardiovascular Surgery, Sechenov First Moscow State Medical University of Health Ministry of Russia, Moscow, Russia.; 2 Department of Cardiac Surgery, Federal Center for Cardiovascular Surgery, Russia; 3 Astrakhan State Medical University, Astrakhan, Russia.; 4 Division of Cardiovascular Surgery, Pronto-Socorro Cardiológico de Pernambuco - Prof. Luiz Tavares, PROCAPE, Recife, Brazil.; 5 University of Pernambuco, UPE, Recife, Brazil.; 6 Nucleus of Postgraduate and Research in Health Sciences of Faculty of Medical Sciences and Biological Sciences Institute, FCM/ICB, Recife, Brazil.

**Keywords:** Heart Valve Diseases, Aortic Valve Diseases, Aorta, Replantation, Prostheses and Implants, Surgeons

## Abstract

In aortic valve disease cases, prosthetic valves have been used for valve replacement, however, these prostheses have inherent problems, and their quality in some countries is lower comparing to new-generation models, causing shorter durability. Aortic valve neocuspidization (AVNeo) has emerged as an option, which can be applied to a wide spectrum of these diseases. Despite the promising results, this procedure is not widely spread among cardiac surgeons yet. We developed a surgical technique combining Bentall and Ozaki procedures to treat patients with concomitant ascending aorta replacement and AVNeo and we describe it in this paper.

**Figure:**
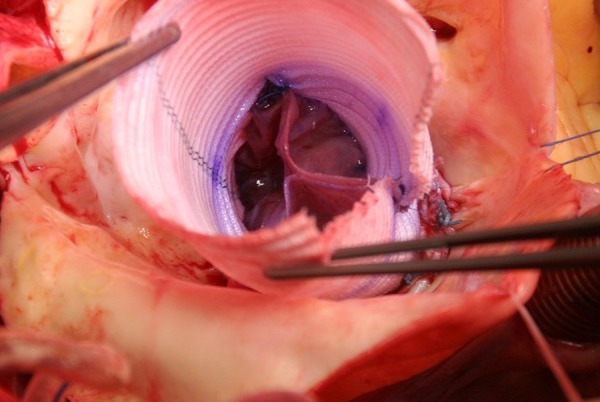
The Russian conduit – combination of Bentall and Ozaki procedures.

The Russian conduit – combination of Bentall and Ozaki procedures.

**Table:** 

Abbreviations, acronyms & symbols	
AA	= Ascending aorta
AV	= Aortic valve
AVA	= Aortic valve annulus
AVNeo	= Aortic valve neocuspidization
DTG	= Dacron tube graft
TEE	= Transesophageal echocardiography

## INTRODUCTION

Bentall-DeBono operation for aortic valve (AV) and ascending aorta (AA) replacement with a valve-containing conduit, first performed in 1966, occupies a unique position in the course of development of operations on the aorta. Currently, it is widely used in cardiac surgery and it is considered as a “gold standard” for pathologies affecting AA and AV^[1,2]^. The advantages of this approach are a proven methodology and high-quality long-term results. The well-known problems associated with this surgical procedure include, among others, complications due to the need to receive anticoagulants when patients choose mechanical prostheses^[3,4]^. Moreover, the use of artificial prostheses has always been associated with a residual pressure gradient, whose severity strongly depends on its type and size and is explained by the presence of a frame and a cuff for fixation, reducing the effective orifice area. Additionally, there is the risk of postoperative infective endocarditis. Bioprostheses, especially in young patients, are prone to structural valve degeneration^[5]^. The exact mechanism through which the fibrosis and/or calcification develops remains undisclosed, being possible the existence of an underlying role of an immune response against the bioprosthesis^[6,7]^.

Ozaki et al.^[8]^ developed a technique for AV cusps replacement, which are cut out according to the original template and made of the patient’s pericardium treated with glutaraldehyde. It is fully logical that, with an aortic aneurysm and degeneratively altered AV cusps, the most promising and most physiologically and surgically convenient treatment option would be a combination of Bentall-DeBono operation with Dacron prostheses and aortic valve neocuspidization (AVNeo) with autologous pericardium (Ozaki procedure).

The objective of this paper is to describe the technique with the Russian conduit for execution of the Bentall-Ozaki procedure, so that other surgeons could become familiarized with this new approach in the treatment of patients with AA disease (aneurysm or dissection) associated with AV disease.

## TECHNIQUE NUMBER 1

### Step 1

**Fig. 1:**
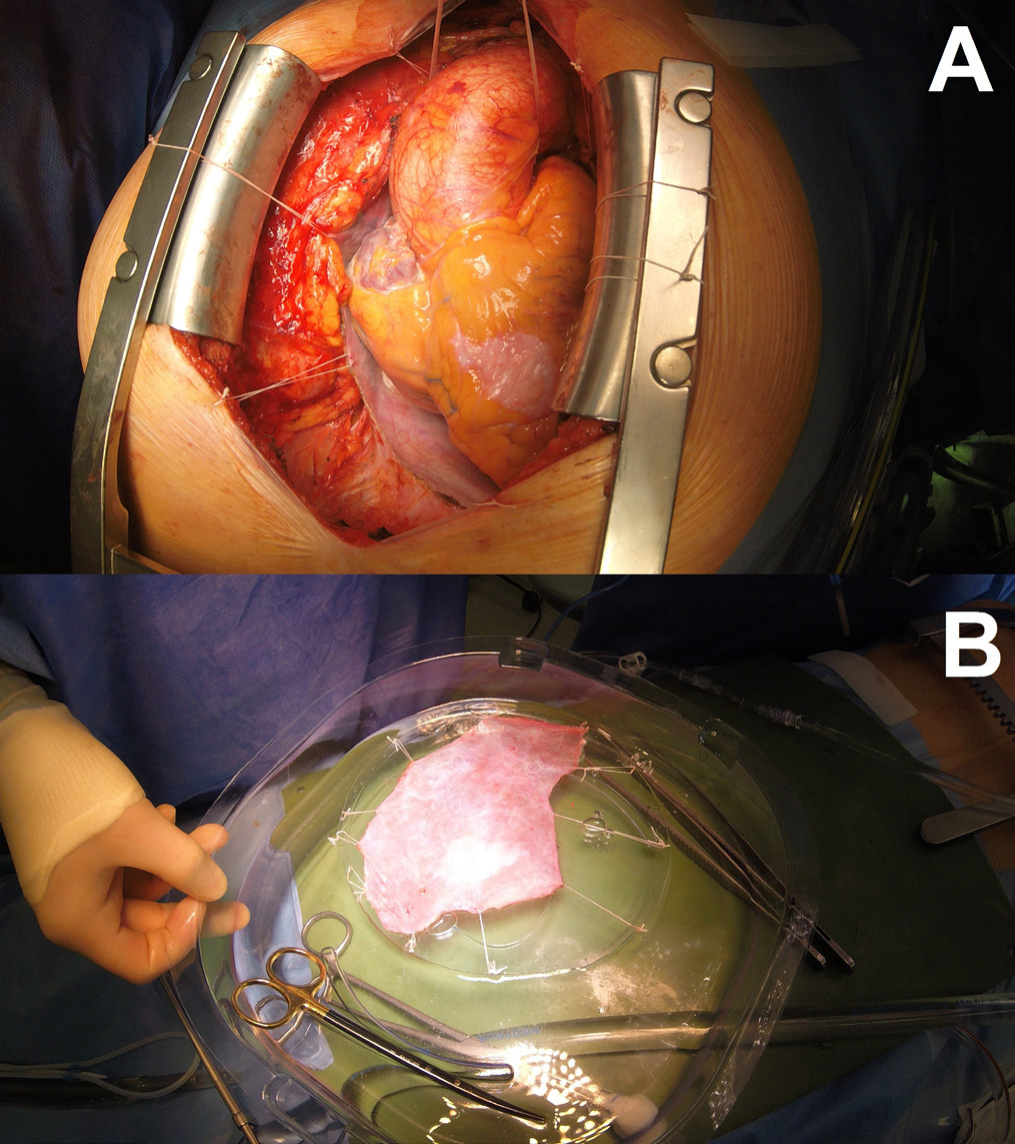
Step 1.

Access to the heart through median sternotomy (Figure 1A) and autologous pericardium harvesting (Figure 1B) with further processing with 0.6% glutaraldehyde for 8 minutes, then treatment twice for 8 minutes with saline solution. Meanwhile, a transesophageal echocardiography (TEE) is performed in order to measure the aortic valve annulus (AVA).

### Step 2

**Fig. 2:**
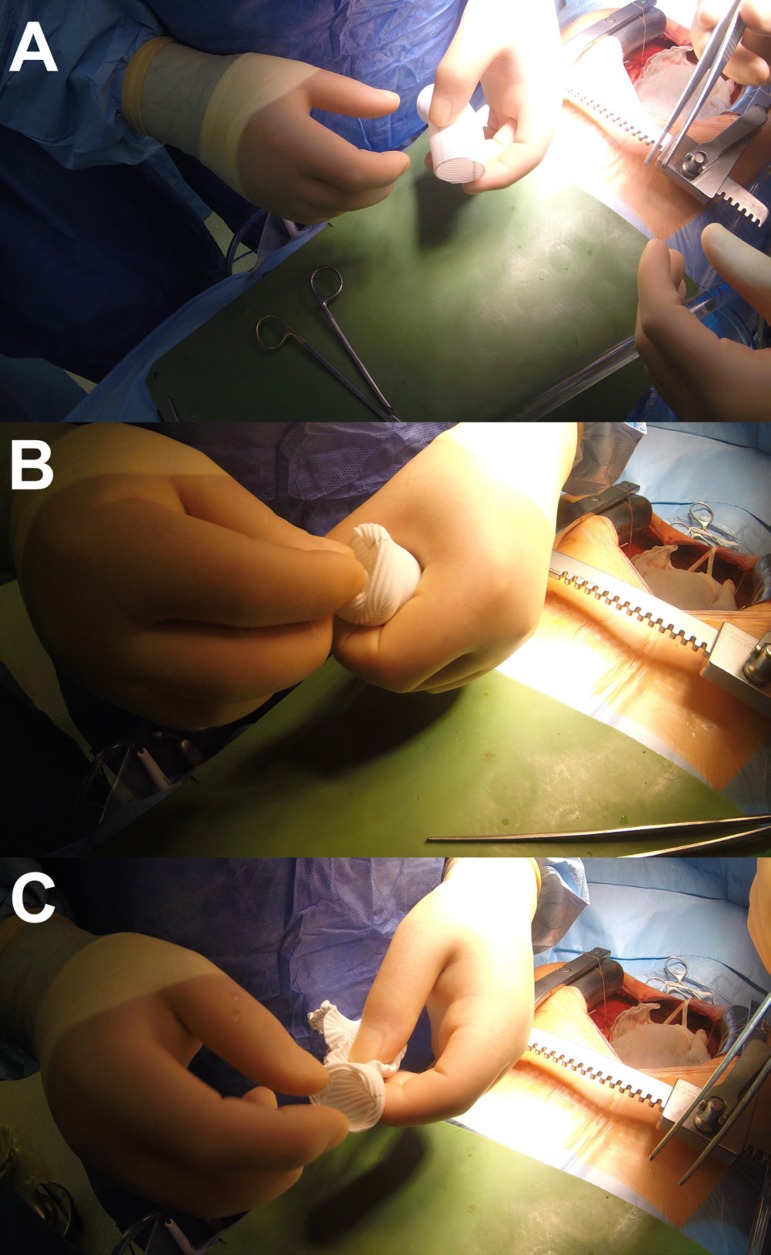
Step 2.

The size of the Dacron tube graft (DTG) must be calculated (Figure 2A). If AVA is not enlarged, then the size of the DTG equals AVA plus 5 mm. If AVA is enlarged, then, 30 or 32 mm. Next, the DTG is everted (Figures 2B and 2C).

### Step 3

Determine the size of the cusps by the following formula:

a). If the size of DTG has an even value, then the size of the neocusps equals DTG’s size minus 1;
b). If the size of DTG has an odd value, then the size of the neocusps equals DTG’s size.

For example, with a DTG of size number 28, we apply the formula ‘28 minus 1 equals 27’. The neocusps’ size corresponds to the number 27 on Ozaki’s template.

### Step 4

**Fig. 3:**
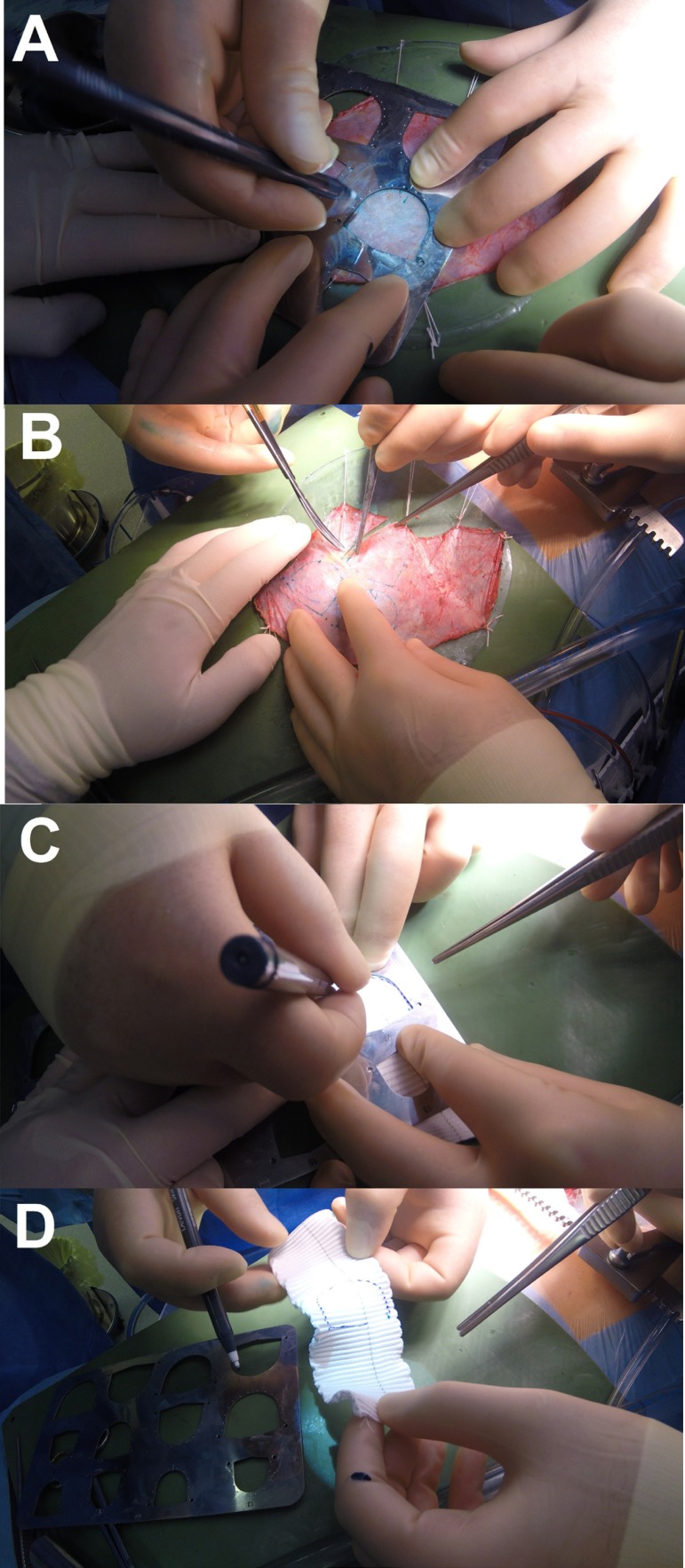
Step 4.

We cut out three identical cusps from the treated autologous pericardium (Figures 3A, 3B, 3C, and 3D)

### Step 5

**Fig. 4:**
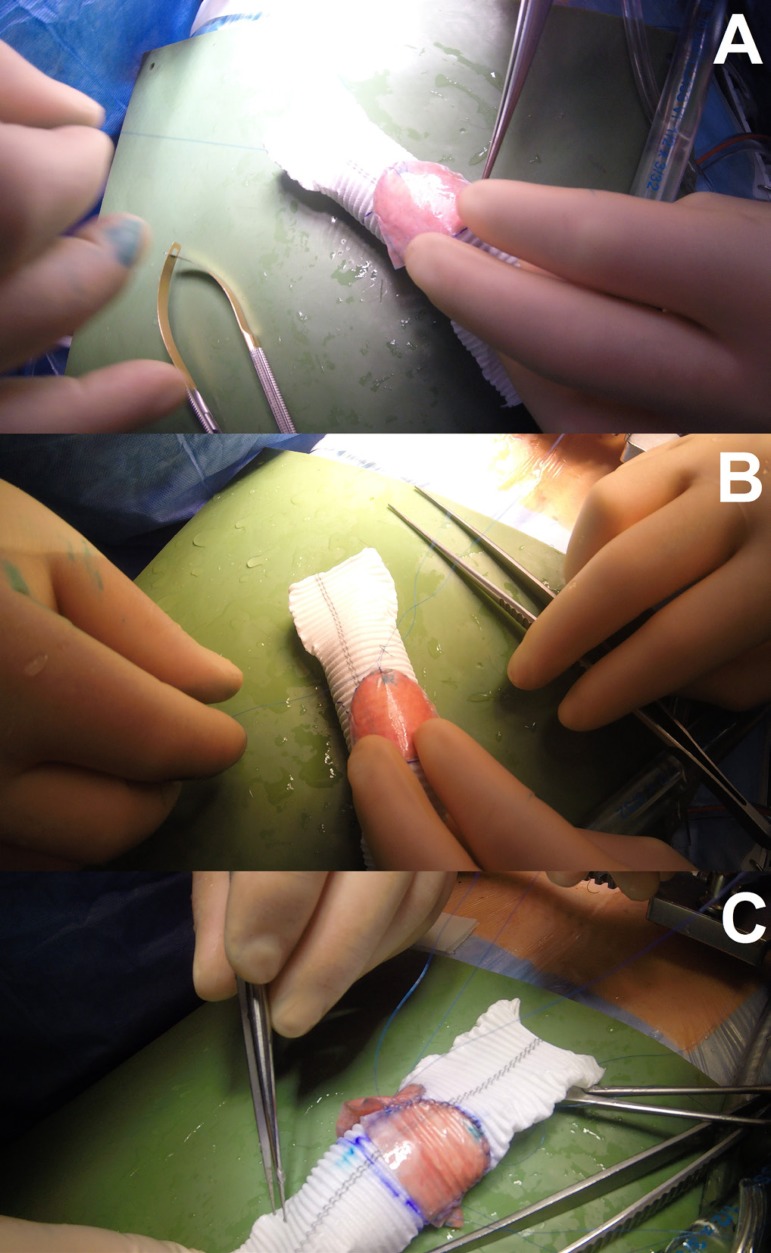
Step 5.

A 5-mm straight line is drawn from the lower edge of the DTG (5 mm will be needed to fix the conduit to AVA). Further, we fix the cusps to the DTG with a continuous suture line with prolene (Figures 4A, 4B, and 4C).

### Step 6

**Fig. 5:**
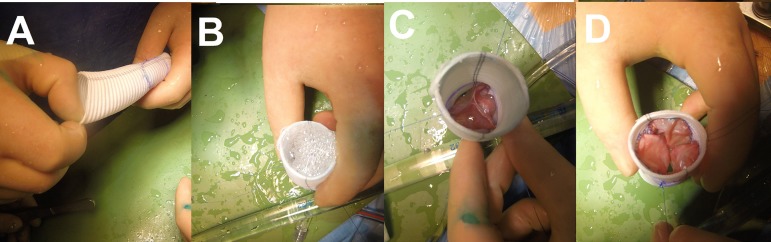
Step 6.

After fixing the cusps, DTG is everted back to its original side (Figures 5A, 5B, 5C, and 5D)

### Step 7

**Fig. 6:**
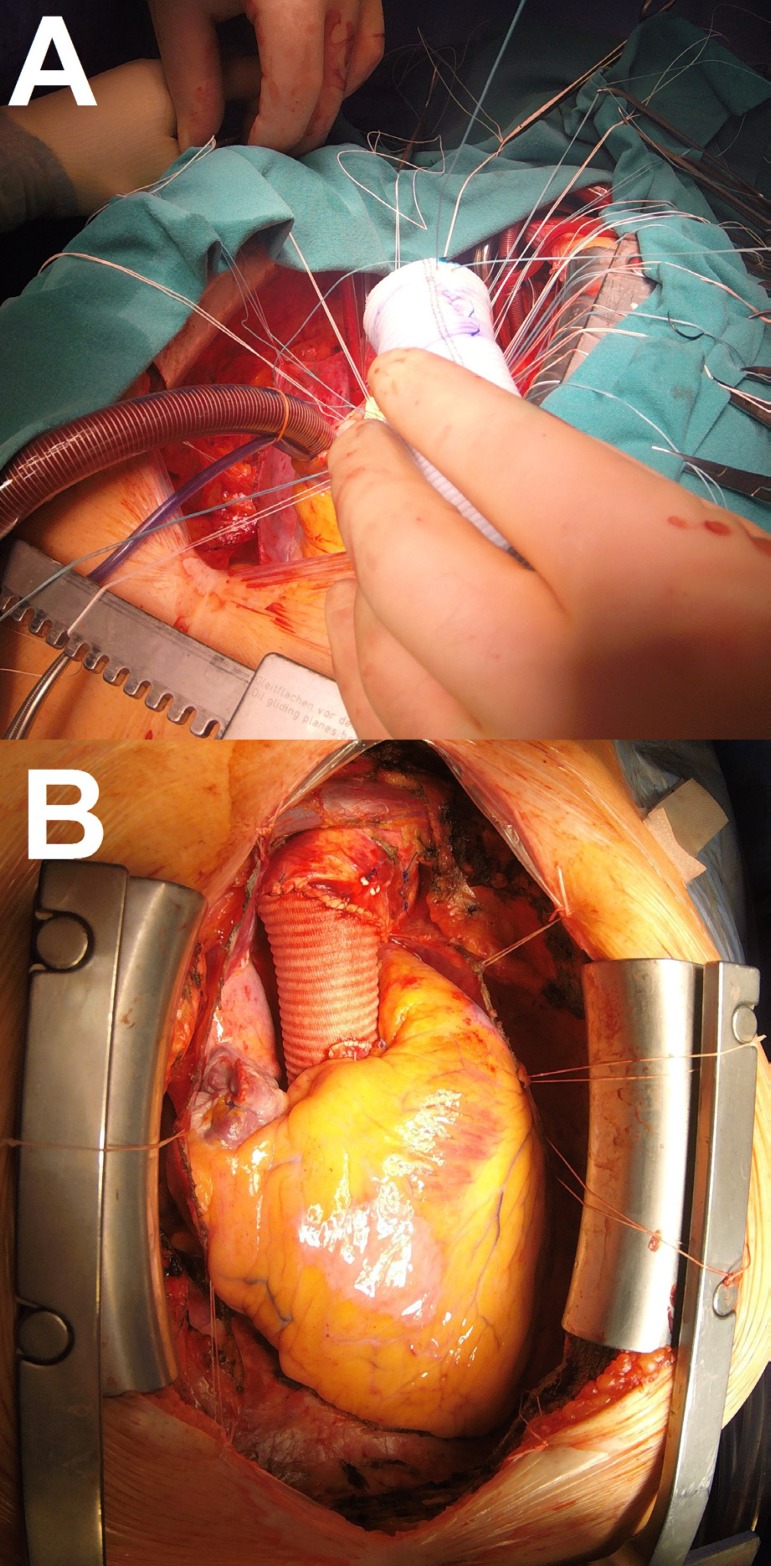
Step 7.

Cardiopulmonary bypass begins, cardioplegic solution is infused, and native AV cusps are excised. The next step is the implantation of the resulting conduit with fixation of the proximal end to AVA with horizontal mattress stitches. Reimplantation of the coronary ostia is performed according to the standard technique. The procedure ends with a distal anastomosis of DTG with AA (Figures 6A and 6B).

## TECHNIQUE NUMBER 2

**Fig. 7:**
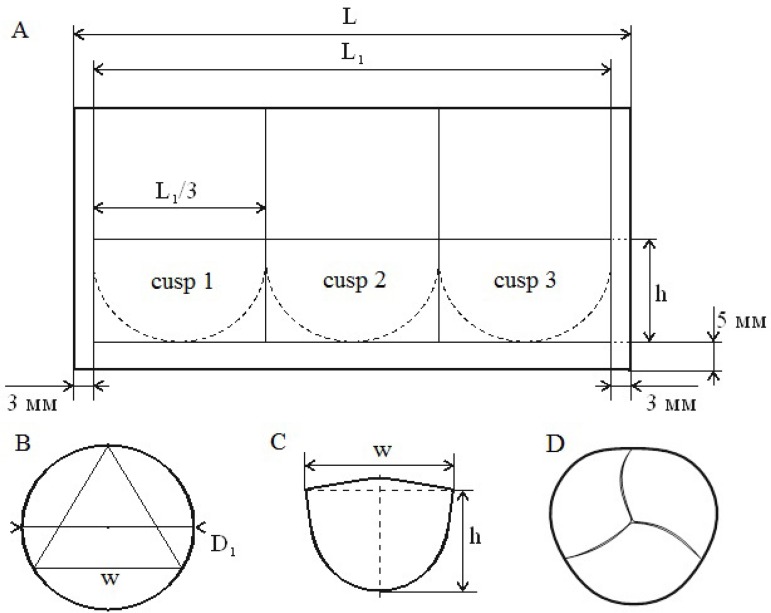
Formulas to calculate the size of the Dacron tube graft (DTG) and of the neocusps.

**Fig. 8:**
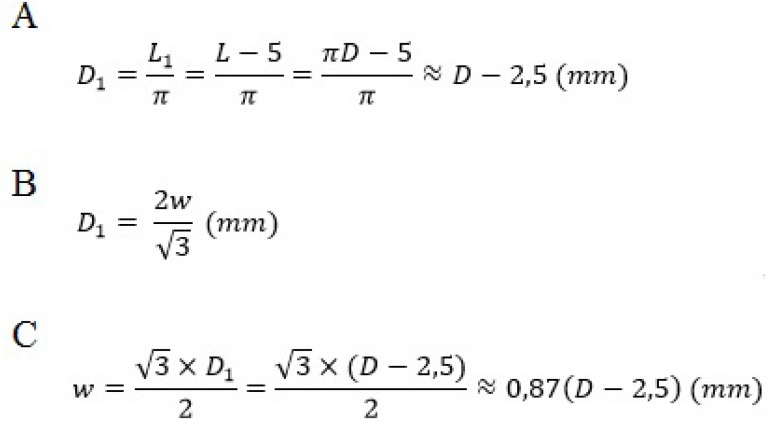
Formulas to calculate the size of the Dacron tube graft (DTG) and of the neocusps.

This technique offers a more elaborate option to calculate the size of the DTG and the size of the neocusps (according to Figures 7 and 8). The diameter of DTG is also based on the previously measured AVA diameter, to which 5 mm is added.

Instead of everting DTG, this is cut along longitudinally, which results in a rectangular section (Figures 7A and 7B) with a width (w) equal to the length (L) of the DTG circumference section, fixed at the angles to a flat surface. Along the left and right edges, straight lines of 2.5-mm size are drawn (Figure 7A) and these are needed for full restoration of the DTG integrity later. Thus, the circumference of DTG (after the later restoration of its integrity) will be L1, which equals to L minus 5 mm, and the diameter of DTG (D1) equals to the formula in Figures 7B and 8A.

**Fig. 9:**
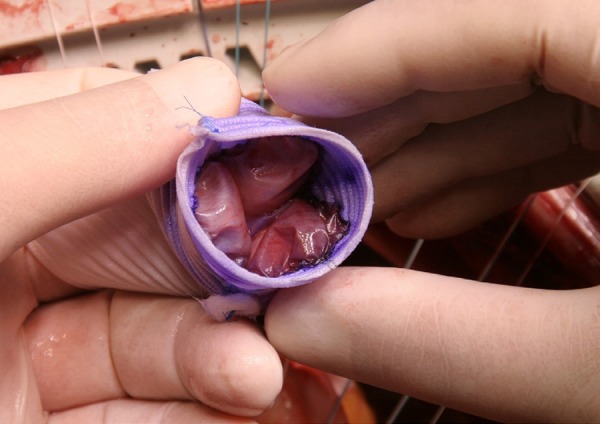
Continuous suture line of prolene is used to fix the neocusps to the Dacron tube graft (DTG).

**Fig. 10:**
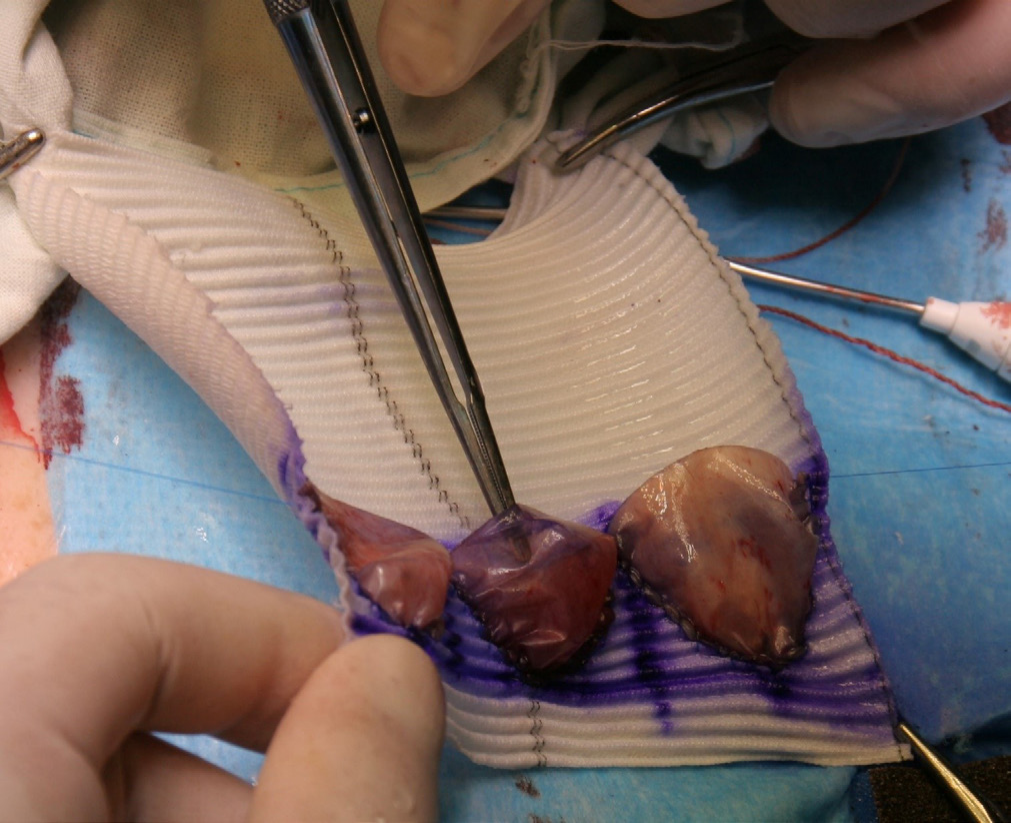
The Dacron conduit is wrapped around itself and restored (as a tube) with a continuous suture line of prolene.

The length of DTG is divided in three equal parts on the back side (Figure 7A). Based on a formula (Figure 8B) for the calculation of the circumference around an equilateral triangle (Figure 7B), we obtain the intercommissural distances (Figures 7C and 8C) and the size of the Ozaki’s template is selected, with the help of which we cut out three identical flaps from the treated autologous pericardium. At 5 mm from the bottom edge of DTG, a straight line is drawn (5 mm will be needed to fix the conduit to the aortic ring). A parallel straight line is also drawn at a distance from the first straight line, equal to the height (h) of the commissures (Figures 7A and 7C), measured according to TEE. This line is then used for fixation of the neocusps to DTG (Figure 7C). Further, a continuous suture line of prolene is used to fix the neocusps to the DTG (Figure 9), which is wrapped around itself and restored (as a tube) with a continuous suture line of prolene (Figures 7D and 10). The next step is the implantation of the resulting conduit with fixation of the proximal end to AVA with horizontal mattress stitches (Central Figure). Reimplantation of the coronary ostia is performed according to the standard technique. The procedure ends with a distal anastomosis of DTG with AA.

## COMMENTS

Most researchers believe that AV prostheses lead to improved longevity, reduce complication rates, and improve quality of life^[9,10]^. The preservation of residual obstruction of blood flow at the level of AV leads to a slower decrease in left ventricular hypertrophy and progression of heart failure^[9-12]^. The use of biological materials for AVNeo is nothing new. Back in the 1960s, the first operations, in which biological materials were used, were performed for AV-sparing procedure with cusp elongation and even completely replacement of the AV cusp^[3]^. The idea of AVNeo using autologous pericardium treated with glutaraldehyde was proposed in 1995 by Duran et al.^[13]^. Gasparyan^[14]^ proposed a formula to calculate the dimensions of the cusps for AVNeo^[14]^. The Ozaki method showed promising mid-term results. After following 416 patients for 73 months, the reoperation-free survival rate was 96.7%^[15]^. There is no doubt that autologous pericardial neovalves have very low thrombogenicity and provide hemodynamics similar to that in the native valve. Also important is the economic aspect, since we can avoid the use of costly prostheses. Further studies are warranted to determine the effectivity of the Russian conduit in the surgical treatment of AV and AA pathologies.

**Table:** 

Authors' roles & responsibilities	
RK	Substantial contributions to the conception or design of the work; or the acquisition, analysis, or interpretation of data for the work; drafting the work or revising it critically for important intellectual content; agreement to be accountable for all aspects of the work in ensuring that questions related to the accuracy or integrity of any part of the work are appropriately investigated and resolved; final approval of the version to be published
IC	Substantial contributions to the conception or design of the work; or the acquisition, analysis, or interpretation of data for the work; drafting the work or revising it critically for important intellectual content; agreement to be accountable for all aspects of the work in ensuring that questions related to the accuracy or integrity of any part of the work are appropriately investigated and resolved; final approval of the version to be published
SE	Substantial contributions to the conception or design of the work; or the acquisition, analysis, or interpretation of data for the work; drafting the work or revising it critically for important intellectual content; agreement to be accountable for all aspects of the work in ensuring that questions related to the accuracy or integrity of any part of the work are appropriately investigated and resolved; final approval of the version to be published
MPBOS	Substantial contributions to the conception or design of the work; or the acquisition, analysis, or interpretation of data for the work; drafting the work or revising it critically for important intellectual content; agreement to be accountable for all aspects of the work in ensuring that questions related to the accuracy or integrity of any part of the work are appropriately investigated and resolved; final approval of the version to be published
DT	Substantial contributions to the conception or design of the work; or the acquisition, analysis, or interpretation of data for the work; drafting the work or revising it critically for important intellectual content; agreement to be accountable for all aspects of the work in ensuring that questions related to the accuracy or integrity of any part of the work are appropriately investigated and resolved; final approval of the version to be published
